# A unique effector secreted by *Pseudozyma flocculosa* mediates its biocontrol activity

**DOI:** 10.1186/s12915-023-01624-z

**Published:** 2023-05-24

**Authors:** Parthasarathy Santhanam, Mst Hur Madina, Fernanda Matias Albuini, Caroline Labbé, Luciano Gomes Fietto, Richard R. Bélanger

**Affiliations:** 1 Département de Phytologie, Université Laval, Québec, QC Canada; 2 Present Address: Agriculture Agri-Food Canada, Morden, MB Canada; 3 Departamento de Bioquímica E Biologia Molecular, Universidade Federal de Viçosa, Viçosa, MG Brazil

**Keywords:** Effectors, Pull-down, CRISPR-Cas9, Hyperbiotrophy, Fungal-fungal interactions, PR proteins, Chitinase, Biocontrol agent, Powdery mildew, *Ustilago maydis*

## Abstract

**Background:**

*Pseudozyma flocculosa* is a highly efficient biocontrol agent (BCA) of powdery mildews whose mode of action remains elusive. It is known to secrete unique effectors during its interaction with powdery mildews but effectors have never been shown to be part of the arsenal of a BCA. Here, we characterize the role of the effector Pf2826 released by *Pseudozyma flocculosa* during its tripartite interaction with barley and the pathogen fungus *Blumeria graminis *f. sp.* hordei*.

**Results:**

We utilized CRISPR-Cas9-based genome editing and confirmed that secreted *P. flocculosa* effector Pf2826 is required for full biocontrol activity. We monitored the localization of the effector Pf2826 with C-terminal mCherry tag and found it localized around the haustoria and on powdery mildew spores. His-tagged Pf2826 recombinant protein was expressed, purified, and used as bait in a pull-down assay from total proteins extracted during the tripartite interaction. Potential interactors were identified by LC–MS/MS analysis after removing unspecific interactions found in the negative controls. A two-way yeast two-hybrid assay validated that Pf2826 interacted with barley pathogenesis-related (PR) proteins HvPR1a and chitinase and with an effector protein from powdery mildew.

**Conclusions:**

In contrast to the usual modes of action of competition, parasitism, and antibiosis ascribed to BCAs, this study shows that effector pf2826 plays a vital role in the biocontrol activity of *P. flocculosa* by interacting with plant PR proteins and a powdery mildew effector, altering the host–pathogen interaction.

**Supplementary Information:**

The online version contains supplementary material available at 10.1186/s12915-023-01624-z.

## Background

The ascomycete fungus, *Blumeria graminis* (DC) Speer f. sp. *hordei* Em. Marchal (Bgh), agent responsible for powdery mildew on barley, is an obligate biotroph that requires a living host to complete its lifecycle. The pathogen invades the host cell and establishes a specialized intercellular feeding structure called haustorium, which plays an important role in assimilating nutrients and delivery of effectors to promote colonization without killing the host [1, 2]. Plant pathogenic fungi secrete effectors that target host proteins to facilitate colonization by suppressing immunity. In the case of Bgh, its genome encodes ~ 6469 genes of which 491 were identified as candidate secreted effectors (CSEPs) and are found only in *Blumeria *spp [3]. Effectors AVR_a1_ and AVR_a13,_ secreted by *B. graminis*, were shown to interact with barley Mla1 and Mla13 to promote immunity [4]. Protein–protein interactions have been studied to discover host targets that are required for resistance. By combining transcriptomic and proteomic data, Pennington et al. (5) showed that a number of Bgh effector candidates (BEC) are secreted in the haustorium, interact with and suppress barley protein(s) for successful infection [5]. Large-scale interactomic data generated by using yeast two-hybrid (Y2H) analysis led to the identification of several *Arabidopsis thaliana* proteins that are targeted by effector proteins from a wide variety of plant pathogenic fungi, oomycetes, and bacteria [6, 7]. Surprisingly, effectors from phylogenetically unrelated pathogens interact with conserved plant targets known as hubs. Highly interconnected hub proteins are involved in defense responses, gene regulation, and primary metabolic processes [8, 9].

*Pseudozyma flocculosa* (Traquair, Shaw & Jarvis) is a powerful antagonist of powdery mildew fungi and appears to target only and specifically members of the *Erysiphales* [10]. Over the years, understanding the exact mode of action of the biocontrol agent has been challenging. Antibiosis, through the secretion of the glycolipid flocculosin, gained a lot of traction initially because of the strong antimicrobial activity of the molecule in vitro [11–14]. Interestingly, a near identical compound, ustilagic acid, was identified in *Ustilago maydis* but the latter failed to exhibit any biocontrol activity against powdery mildews [15, 16]. Attempts to functionally characterize the role of flocculosin over the years have been hampered by the inability to transform *P. flocculosa* through homologous recombination [10].

Using CRISPR-Cas9, Santhanam et al. (17) were recently able to successfully knock out *Fat1*, a gene required for the biosynthesis of flocculosin. The resulting knock out strain was unable to produce flocculosin, yet its biocontrol activity on powdery mildew colonies was similar to the wild type [17]. This offered strong evidence that flocculosin-mediated antibiosis was not how *P. flocculosa* exerted its biocontrol activity, a conclusion that had been previously suggested by Laur et al. (18) who had completed a transcriptomic analysis of the tritrophic interaction *P. flocculosa*-barley-powdery mildew [18]. Indeed, the latter group reported that *P. flocculosa* secreted several unique effector proteins exclusively during its interaction with Bgh on barley. As stated earlier, pathogens secrete effectors to manipulate plant defense mechanisms for successful infection but the notion that a biocontrol agent (BCA) would employ a similar strategy to attack a pathogen remains speculative and unproven. Earlier, Laur et al., based on the observation that some effectors were upregulated nearly 2000 times more in presence of Bgh than in vitro, proposed that *P. flocculosa* destabilized the biotrophic state of Bgh through the release of effectors that exacerbated the production of haustorial effectors [18]. However, the authors could not assess a role or localization of *P. flocculosa* effectors or whether they interacted with barley or Bgh proteins.

The main objective of this study was to functionally characterize and establish the role played by Pf2826, a unique effector released by *P. flocculosa* during the tripartite interaction with barley and Bgh. For this purpose, we used CRISPR-Cas9 technique to generate knockouts of *Pf2826* and evaluate the biocontrol activity of mutant strains. Moreover, subcellular localization of effector Pf2826 during the tripartite interaction was carried out using C-terminal mCherry tag. To identify, the interacting partners of Pf2826, we employed pull down assays followed by mass spectrometry and validated the interactions by a two-way Y2H approach. The functional analysis confirms that the effector Pf2826 is essential to the biocontrol activity of *P. flocculosa* on powdery mildews, which represents an unusual and unprecedented mode of action for a BCA.

## Results

### Reduction of biocontrol activity through knockout of effector Pf2826

Effector Pf2826 was selected for knockout based on the report by Laur et al. [18] showing its high expression only during the tripartite interaction. The effector Pf2826 is 454 amino acids long and predicted to contain an N-terminal signal peptide. Interpro scan of Pf2826 resulted in the identification of two LVIVD repeats (IPR013211), which are commonly found in bacterial and archeal cell surface proteins with unknown functions. Blast search of Pf2826 against non-redundant protein sequence (nr) database showed that Pf2826 was unique, and the closest relative was found in a lichen (*Caloplaca aetnensis*) with 59.2% identity. To knockout *Pf2826* using CRISPR-Cas9, a 20 nt crRNA was designed starting at the 173rd nucleotide from the start codon (Additional file 1: Fig. S1A). To monitor the cleavage efficiency, the coding sequence of *Pf2826* (1.4 kb) was PCR amplified and mixed with Cas9 nuclease complexed with freshly prepared sgRNA. Results showed that crRNA-173 was able to successfully cleave *Pf2826* gene under in vitro conditions (Additional file 1: Fig. S1B) and it was further used for RNP-mediated Cas9 delivery.

**Fig. 1:**
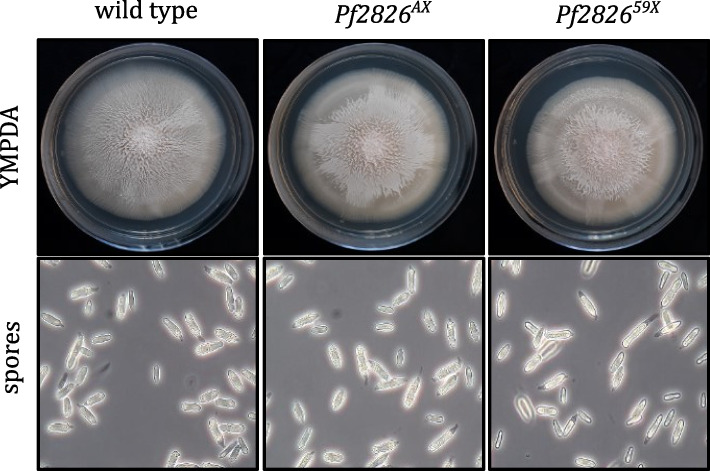
Knocking out *Pf2826* has no effect on the radial growth or spore production. Radial growth and spore production were compared between wild type *Pseudozyma flocculosa* and Cas9 knock-out strains *Pf2826*^*AX*^ and *Pf2826*^*59X*^. Radial growth was assessed on YMPDA (7 dpi). Spore production was monitored by growing the strains in YMPD liquid culture (3 dpi). The experiment was repeated three times with similar results

**Fig. 2:**
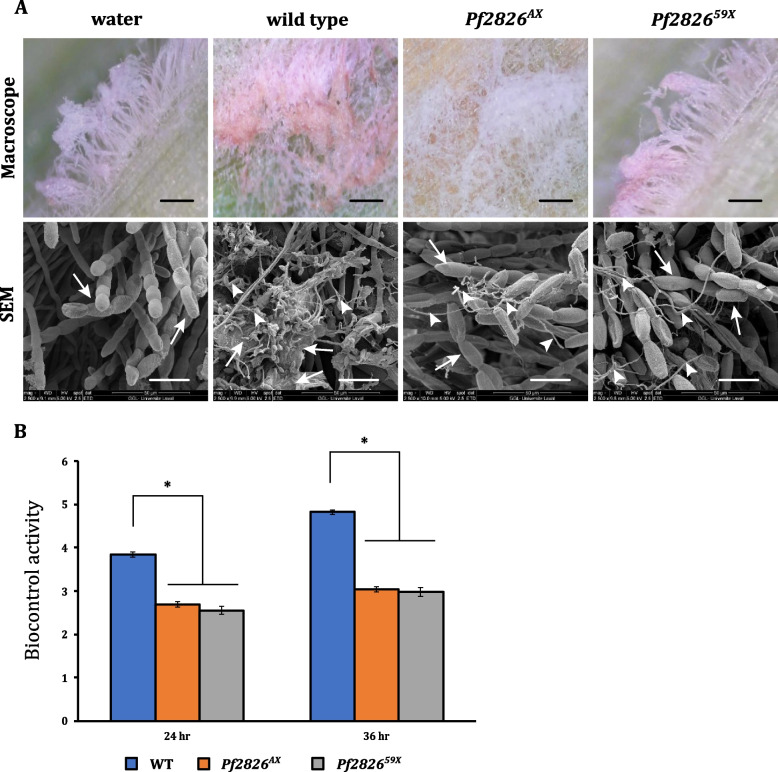
Knocking out *Pf2826* resulted in loss of biocontrol activity. Biocontrol activity of wild type *Pseudozyma flocculosa* and Cas9 knock-out strains *Pf2826*^*AX*^ and *Pf2826*^*59X*^ were tested against *Blumeria graminis *f. sp.* hordei* (Bgh) over time. **A** Stereomicroscopy and scanning electron microscopy (SEM) observations of the biocontrol activity against Bgh at 36 h post-inoculation (hpi). Arrows show Bgh conidia; arrowheads show *P. flocculosa* structures. **B** Biocontrol activity was rated on a scale of 0 to 5 where 0 = no activity and 5 = complete collapse of powdery mildew colonies at 24 and 36 hpi. Each value is the mean of three independent experiments ± standard error (SE). Each experiment comprised five leaves and three different sites per leaf. *p < 0.01; indicates significant difference by Student’s *t* test (Additional file 6). Black bars = 400 μm; white bars = 25 μm

Genomic DNA was isolated from the selected potential mutants and used as a template to amplify a 125-bp region flanking the sgRNA binding site. The amplicon was subjected to high-resolution melting (HRM) analysis, and fluorescence difference curve was used to identify the mutants (Additional file 1: Fig. S2). To verify the mutations predicted by HRM analysis, the full-length coding sequence of *Pf2826* was PCR amplified and sequenced. Following delivery attempts on *P. flocculosa* sporidia using CRISPR-Cas9, two positive mutants were confirmed: one with a deletion of one adenosine nucleotide, and the other with a deletion of 59 nucleotides (Additional file 1: Fig. S3A). As a result, the reading frame was modified in each strain resulting in an altered and shorter protein (Additional file 1: Fig. S3B). The mutants were identified as *Pf2826*^*AX*^* and Pf2826*^*59X*^, respectively. When compared to the wild type, Cas9 mutants *Pf2826*^*AX*^ and *Pf2826*^*59X*^ did not show any difference in growth or in spore production under in vitro conditions (Fig. 1). On the other hand, when *Pf2826*^*AX*^* and Pf2826*^*59X*^ mutant strains were tested for biocontrol activity against powdery mildew on barley, they developed significantly slower and less aggressively (Fig. 2A) and never succeeded to overtake the powdery mildew colonies even at 36 hpi (Fig. 2B).

### Localization of the effector Pf2826 during tripartite assay

**Fig. 3:**
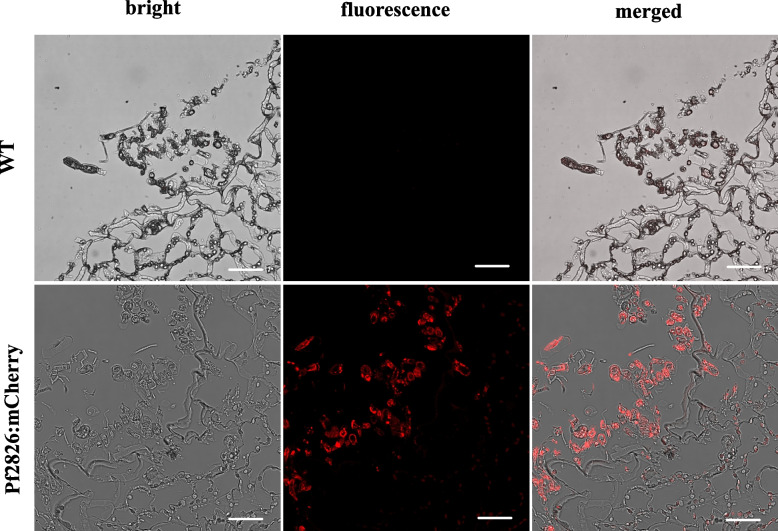
Subcellular localization of the effector Pf2826 during tripartite assay. The Pf2826 tagged with mCherry was introduced into wild type *Pseudozyma flocculosa*, and the transformant was inoculated on barley leaves infected with powdery mildew. Wild-type *P. flocculosa* without fluorescence tag was used as control. Samples were collected 24 hpi, sectioned, and observed under Leica SP8 confocal microscope. Bars = 20 μm. Raw Z-stack images are provided as a short video (Additional file 2 and Additional file 3)

We examined the subcellular localization of Pf2826 using confocal microscopy. To visualize Pf2826 movement, we made a construct encoding the mature Pf2826 protein that was C-terminally fused to a red fluorescent protein (mCherry) under the control of constitutive promoter of translation elongation factor 3 (eEF3). Positive transformants expressing bright red under in vitro conditions were selected and used for the tripartite assay on barley infected with powdery mildew. Sectioned samples from the tripartite interaction were observed under a confocal microscope to monitor red fluorescence. As expected, no red fluorescence was observed in wild type-inoculated leaves except for autofluorescence from chloroplasts (Fig. 3). However, in leaves inoculated with the strain expressing Pf2826:mcherry, a strong red fluorescence signal was clearly observed within the haustorium and inside and around powdery mildew conidia (Fig. 3; Additional file 2 and Additional file 3). While the fluorescence appeared concentrated along the cell wall of the conidia on the leaf surface, its presence in the haustorium indicates that, if there is interaction with targets in the cell wall, it is not the only substrate with which the effector would interact given the absence of plant cell-wall components in the haustorium.

### In vitro pull-down assay to identify potential interactors of Pf2826

In vitro pull-down and co-immunoprecipitation (Co-Ip) approaches are commonly used to identify stable protein–protein interactions. It is feasible to clone, express, and purify the bait protein for pull-down assays. In the case of Co-Ip, specific antibodies against the bait (Pf2826) need to be generated, an expensive and time-consuming approach where success mainly depends on an effective tertiary structure prediction. For this reason, an in vitro pull-down assay was performed to determine whether proteins from Bgh and/or barley interacted with the highly secreted *P. flocculosa* effector Pf2826. Based on SDS-PAGE, we observed many proteins eluted in all three pull-down assays. Interacting proteins were identified by in-gel digestion followed by LC–ESI–MS/MS analysis. Potential interactors that are unique only to the Pf2826 (79 proteins) were identified after removing the proteins present in negative controls (Additional file 4: Table S1). Initial observations revealed the bulk of the interacting proteins came from barley (52 proteins), followed by Bgh (20 proteins) and finally by *P. flocculosa* with only seven proteins. More than 25% of potential interactors were membrane proteins (20), and around 20% were ribosomal proteins (15). Gene Ontology (GO) term annotation associated with 79 candidate proteins were predicted using Blast2GO and classified into three categories (molecular function, cellular components, and biological process). The majority of the potential interactors of Pf2826 were involved in cellular (27%) and metabolic (24%) processes. Proteins involved in response to stimulus (4%) and signaling (2%) were also found (Additional file 1: Fig. S4). Based on their localization and predicted biological functions, eight candidate targets were selected as potential interactors (Additional file 5: Table S2).

### Two-way Y2H to validate interactors

**Fig. 4:**
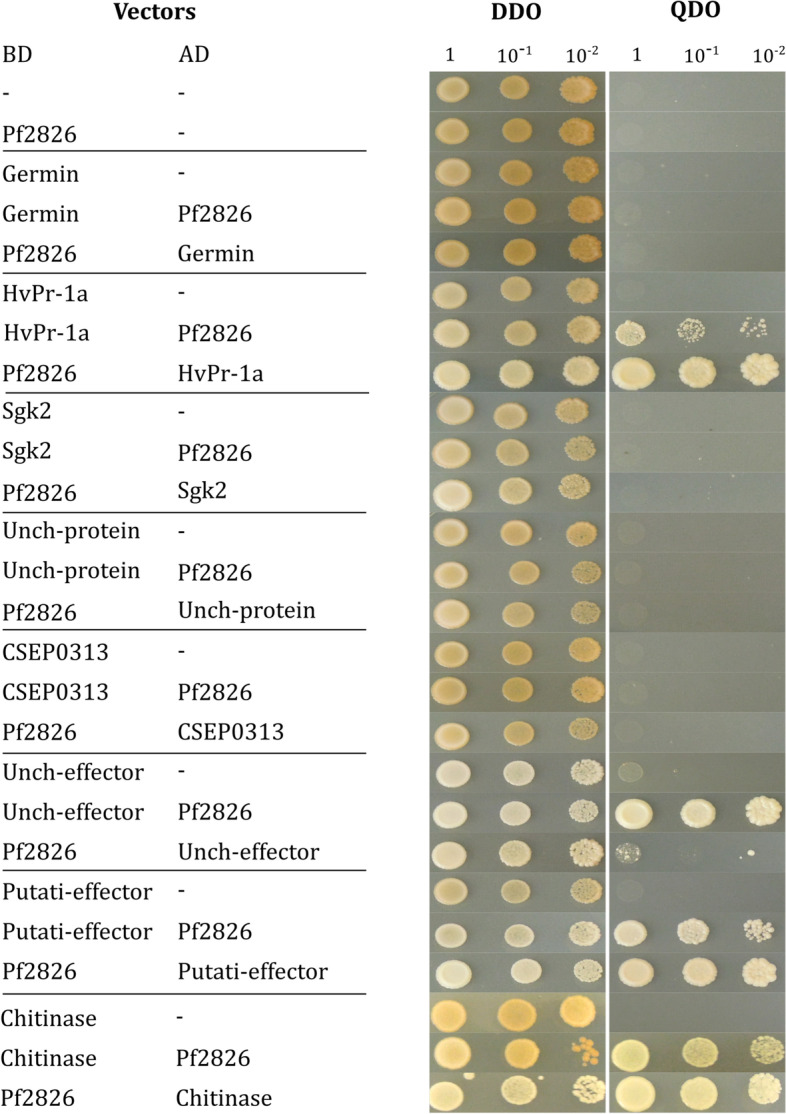
Yeast-2-hybrid assays showing the interactions between effector Pf2826 from *Pseudozyma flocculosa* and candidate proteins from barley and *Blumeria graminis* f.sp. *hordei*. Interactions were first named for the bait (BD vector) followed by the prey (AD vector). From top to bottom, candidate interactors are A0A287N0S4 (germin); A0A287RAV7 (HvPR-1a); N1J4Z2 (Sgk2), A0A383V2V0 (Unch-Protein), N1J6Z3 (CSEP0313), A0A383UML4 (Unch-effector), N1J908 (Putati-effector), and F2DJR4 (Chitinase). Log-phase cultures of Y2HGold yeast cells containing plasmids with different combinations were washed and plated at different dilutions on non-selective media (lacking tryptophan and leucine) and selective media (lacking tryptophan, leucine, histidine, and adenine) and incubated at 30 °C for 3 to 5 days. Growth promotion on selective media indicates interaction. Growth in bi-directional cloning indicates strong interaction, while growth in one cloning direction indicates weak interaction. Results shown are representative of those obtained from three independent transformants

Eight potential interactors of the Pf2826 effector identified by the pull-down assays were selected for validation by the two-way Y2H method. The eight selected candidates were A0A287N0S4 (germin); A0A287RAV7 (HvPR-1a) and F2DJR4 (Chitinase) from barley, N1J4Z2 (Sgk2), A0A383V2V0 (Unch-Protein), N1J6Z3 (CSEP0313), A0A383UML4 (Unch-effector), and N1J908 (Putati-effector) from Bgh. The selection of the targets from barley and powdery mildew was based on their molecular function and cellular localization. All selected targets from barley are potentially involved in stress responses and presented predicted signal peptides, which indicates that they are secreted to the extracellular region. Three out of the five targets from Bgh were identified as candidate secreted effector proteins (N1J6Z3, A0A383UML4 and N1J908). In addition, Bgh potential interactors included a serine/threonine-protein kinase (N1J4Z2) and an uncharacterized protein related to the COP9 signalosome complex (A0A383V2V0). The Pf2826 effector and targets were co-transformed into the Gold Yeast Two-Hybrid strain using pGADT7 and pGBKT7 plasmids. The interaction was evaluated using selective media (QDO). Empty plasmids (pGBKT7 and pGADT7) were used as negative controls. The growth on QDO indicated that the Pf2826 effector interacted in yeast with HvPR-1a (A0A287RAV7) and a chitinase (F2DJR4), both from barley, and also with a candidate secreted effector protein (N1J908) from Bgh in both bait-prey cloning orientations (Fig. 4). Yeast lines expressing the uncharacterized effector protein (A0A383UML4) from powdery mildew predominantly grew in one bait-prey orientation on selective media, indicating weak interactions. The yeast lines expressing the other target proteins behaved like the negative controls and did not grow on selective media (QDO), which indicated a lack of interaction between these prey proteins and the Pf2826 effector (Fig. 4).

### Overexpression of Pf2826 in *U. maydis*

**Fig. 5:**
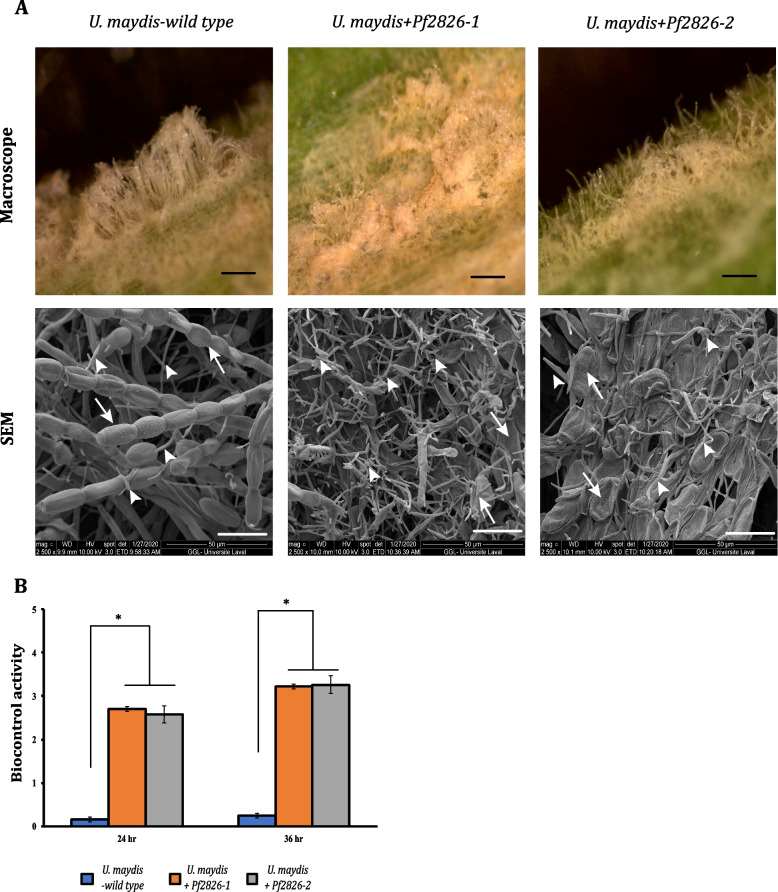
Overexpression of *Pf2826* transforms *Ustilago maydis* (FB1) into a biocontrol agent. Barley plants infected with *Blumeria graminis* f.sp. *hordei* (Bgh) were inoculated with *U. maydis* wild-type strain FB1 and two independent FB1 transformants overexpressing *Pf2826* strains. **A** Stereomicroscopic and scanning electron microscopy (SEM) observation of biocontrol activity of wild type and overexpression strains at 36 hpi. Arrows show Bgh conidia; arrowheads show *U. maydis* structures. Black bars = 400 μm; white bars = 25 μm. **B** Biocontrol activity was rated on a scale of 0 to 5 where 0 = no activity and 5 = complete collapse of powdery mildew colonies at 24 and 36 hpi. Each value is the mean of three independent experiments ± standard error (SE). Each experiment comprised five leaves and three different sites per leaf (Additional file 7). *p < 0.01; indicates significant difference by Student’s *t* test

Considering the close phylogenetic link between *P. flocculosa* and *U. maydis*, we exploited the opportunity to further validate the role of *Pf2826* by expressing the gene in the latter fungus. Positive *U. maydis* transformants growing on medium supplemented with carboxin were selected and checked for the expression of Pf2826 under in vitro conditions. Two highly expressing *U. maydis* + Pf2826 strains were selected for assessment of biocontrol activity on barley leaves infected with Bgh. Wild type *U. maydis* showed limited development on Bgh colonies with no apparent or minimal signs of biocontrol activity. On the other hand, both *U. maydis* strains expressing *Pf2826* displayed strong and extensive growth on Bgh colonies resulting in their complete collapse in a manner similar to wild type *P. flocculosa* (Fig. 5).

## Discussion

Upon providing the full genome sequence of *P. flocculosa*, Lefebvre et al. identified several new putative genes that could explain the unique activity of the biocontrol agent [19]. Among them, the presence of 200 unique genes coding for candidate secreted effector proteins raised questions about the evolutionary importance of maintaining such traits. In a transcriptomic study looking at the gene expression of all three actors of the tripartite interaction *P. flocculosa*-*B. graminis*-barley, Laur et al*.* reported the high expression of a number of effector genes concomitant with the biocontrol activity of *P. flocculosa* [18]. This brought a novel hypothesis into consideration whereby a unique effector released by *P. flocculosa* would facilitate its antagonism toward powdery mildews. In this work, we present evidence that this effector released by the BCA, *P. flocculosa*, plays a functional role in the biocontrol activity of *P. flocculosa* against powdery mildews by interacting with proteins belonging likely to both the plant and the pathogen.

While effector proteins are known to play a major role in plant pathogen-interactions, the notion that an effector would dictate the activity of a BCA represents a novelty. To test this hypothesis, we focused on effector *Pf2826*, owing to its high and exclusive expression during the tripartite interaction [18] and altered its production using CRISPR-Cas9. Our results clearly showed that resulting mutant strains significantly lost biocontrol activity on *B. graminis *f. sp.* hordei* yet maintained wild-type-like growth and sporulation. Interestingly, the same phenotype was observed with two independent transformants with different nucleotide deletions indicating that the phenotype was associated with the expression of *Pf2826*. Moreover, due to the short lifespan of the RNP complex, the possibility of off-target mutations can be ruled out [20]. This represents the first instance linking a functional effector with a BCA, an observation that raises many questions about this unusual role for an effector.

As a first point of inquisition, the localization of the effector, once released, was of particular interest given that two potential hosts are in play. For this purpose, we decided to overexpress Pf2826 tagged with mCherry using a constitutive promoter (eEF3). The Pf2826:mCherry was found to be localized within the haustoria and in and around Bgh spores. This would support a rare instance where a fungus-fungus interaction is mediated by an effector. However, the interactions may be more complex than anticipated. Indeed, the haustorium is a well-known interface structure established by the invading biotrophic fungi for the uptake of nutrients and to suppress host defense responses [2]. It is also an active site for the release of effector proteins from the pathogen into the plant tissue, as the secreted effectors are in proximity with the plant cell membrane [2]. As a result of the extrahaustorial matrix being composed of both fungal and plant elements, there is the possibility that Pf2826 also or exclusively interacts with plant proteins.

**Fig. 6:**
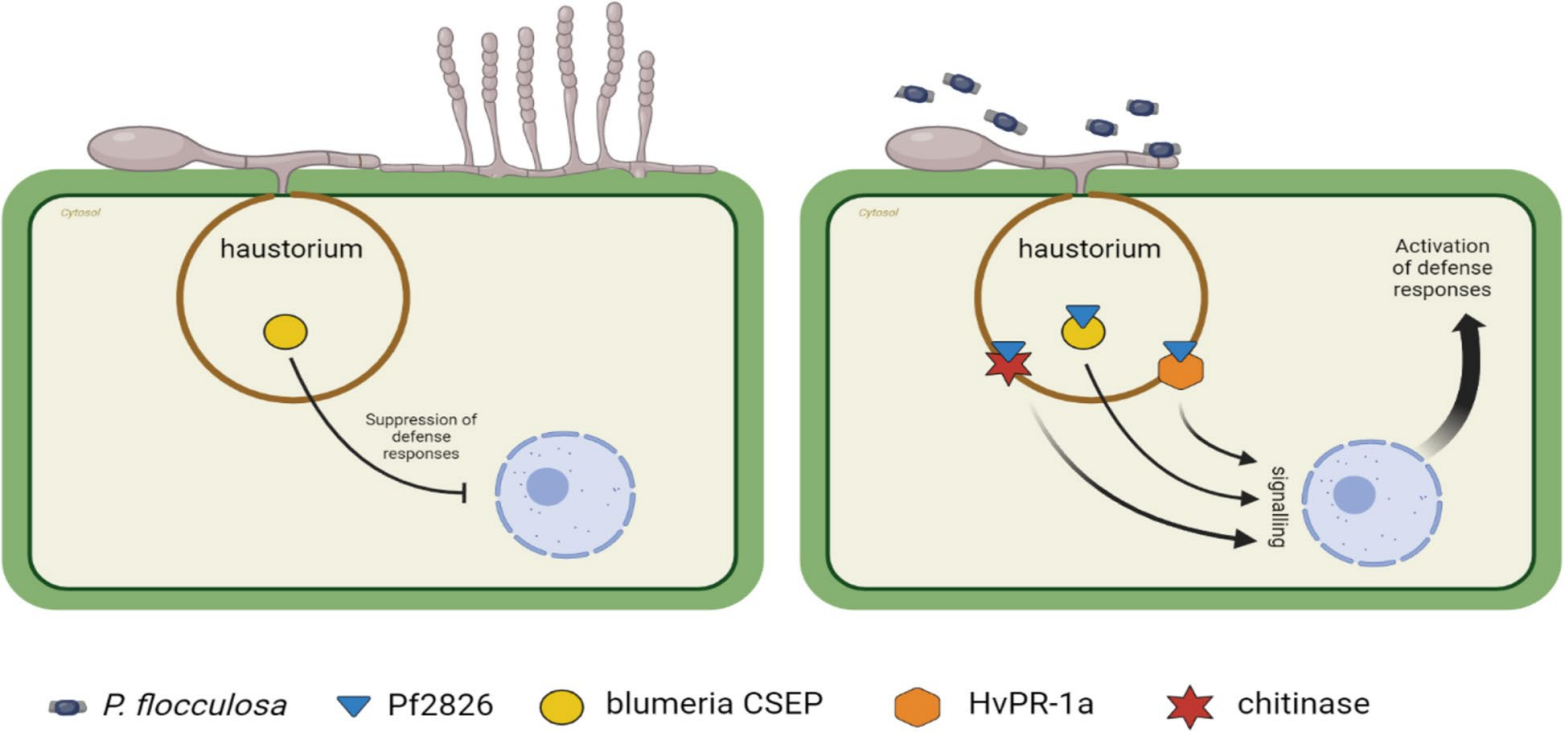
Hypothetical model for the role of Pf2826 in the biocontrol activity of *Pseudozyma flocculosa*. *Blumeria graminis *f. sp.* hordei* (Bgh) successfully supresses host immunity, colonizes, and causes disease on barley. When *P. flocculosa* is inoculated on Bgh- infected barley, Pf2826 is secreted and translocated to Bgh spores and the haustorium region of barley epidermal cells. Once translocated, Pf2826 interacts with Bgh candidate effector protein (CSEP) and barley HvPR-1a and chitinase. This interaction destabilizes Bgh-induced immunity suppression, resulting in the activation of an immune response and collapse of haustoria and powdery mildew colonies

In an attempt to determine the potential interactors with Pf2826, we used a pull-down assay followed by MS/MS analysis for preliminary identification and validated them by targeted two-way Y2H assay. Both approaches are complementary with respect to the interactors they can detect. The pulldown assay is an in vitro methodology that can identify many molecules that constitute larger complexes, even though not all proteins interact directly with the bait. The Y2H assay is an in vivo system that evaluates the interaction between two proteins in yeast. The heterologous expression of proteins in yeast may hinder proper post-translational modifications and, consequently, negatively affect the interaction [21]. It is relevant to highlight that the combination of these two approaches increases the chance of identifying a genuine interaction. In our study, Pf2826 interacted with three proteins in both assays: a pathogenesis-related (PR) protein-1a (HvPR-1a) from barley, a chitinase from barley and a putative secreted effector from Bgh (Fig. 4). Pathogenesis-related proteins are known to reduce virulence, pathogen growth, and colonization [22, 23]. Pennington et al. [5] showed that Bgh effectors interacted with PR proteins and inhibited their antifungal activity [5]. Interestingly, both HvPR-1a (A0A287RAV7) and chitinase (F2DJR4) were among the top 10 highly expressed barley genes post *P. flocculosa* inoculation [18]. These results suggest that the effector Pf2826 could interact with the host defense-related targets resulting in the elicitation of host defense mechanisms. On the other hand, Pf2826 also interacted with Bgh putative secreted effector (N1J908), which shares a high homology (87%) with CSEP0128. Aguilar et al. [24] showed that CSEP0128 was essential for early aggressiveness of Bgh and that knockout of the effector resulted in reduction in haustorium formation. Therefore, the interaction of Pf2826 with N1J908 might also play a role in altering the balance the haustoria have established with plant cells. Altogether, the interaction between the Pf2826 and barley PR proteins and a Bgh effector protein are consistent with the phenotype of the interaction showing a deregulation of the delicate biotrophic interaction between barley and Bgh. As schematically illustrated in Fig. 6, *P. flocculosa* effector Pf2826 interacts with barley HvPR-1a and chitinase and with a Bgh candidate effector protein. This interaction would destabilize the delicate Bgh-barley interaction through an activation of defense reactions as suggested by Laur et al. [18] and a concomitant effect on the haustorium resulting in collapse of the latter and Bgh colonies (Fig. 6). In this work, we provide direct evidence that effector Pf2826 plays an important role in the biocontrol activity of *P. flocculosa*. However, apart from Pf2826, other effectors are secreted by *P. flocculosa* during the tripartite interaction and their cumulative impact may influence the activity of the BCA as it is well known that effectors have multiple targets.

The well-studied basidiomycete pathogen *Ustilago maydis*, closely related to *P. flocculosa* [19], secretes ustilagic acid, a molecule similar to flocculosin that possesses broad spectrum antifungal activity [25]. This unusual link has been at the center of controversies regarding the role and the necessity of the trait in the evolutive process of the two fungi [16, 26]. It presented therefore a good opportunity to use *U. maydis* as a surrogate expressor of Pf2826 to further study the role of the effector. Our results confirmed that wild type *U. maydis* had no effect on powdery mildew but offered clear evidence that *U. maydis* isolates expressing *Pf2826* displayed biocontrol activity against Bgh. This represents a distinctive case of altering a fungus with a single trait to confer biocontrol activity and offers direct proof that Pf2826 is a bona fide determinant of *P. flocculosa* biocontrol properties.

To our knowledge, our results highlight a unique occurrence where an effector protein plays a direct role in the biocontrol activity of a fungus. As there is no precedent for an effector secreted from a fungal biocontrol agent influencing the activity of a pathogenic fungus, it is rather difficult to speculate on the type of protein–protein interaction that could derail the biotrophic interaction between a plant and a powdery mildew fungus. Based on Laur et al*.*’s observations, the main transcriptomic changes occurred between 12 and 24 h after *P. flocculosa* application and were characterized by a transient increase in expression of haustorial effector genes by the pathogen which coincided with a higher metabolic activity [18]. This in turn led to the collapse of haustoria, suggesting that the delicate balance maintaining the state of biotrophy had been disrupted. The possibility that Pf2826 and other *P. flocculosa* effectors interacted with plant receptors that enacted a defense response by the plant should also be considered. Based on our pull-down assay, results suggest that *P. flocculosa*’s effector could interact with either powdery mildew or barley, or maybe both. This interaction leads to a destabilization of the delicate balance of nutrient absorption from the haustorium while keeping the cell alive ending with the collapse of the powdery mildew colonies (biocontrol activity). In earlier works, it was assumed that flocculosin released by *P. flocculosa* first caused the collapse of the ectotrophic mycelium, which in turn would lead to the collapse of the haustorium. Based on the recent report by Santhanam et al. (2022) showing that flocculosin is not involved in the biocontrol activity of *P. flocculosa* and results reported here showing clearly that the effector is translocated around the haustorium, it is now more likely that the haustorium is the first point of attack. As a matter of fact, these findings are congruent with previous observations, notably that the interaction is strictly dependent on the presence of the haustorium inside the plant, since preventive applications of *P. flocculosa* were never shown to alter powdery mildew infections and that biocontrol activity of *P. flocculosa* appears to be exclusively directed against powdery mildews [15], since applications against necrotrophic fungi were never successful. On the other hand, our results raise the intriguing question whether overexpression of *Pf2826* in plants would provide durable resistance against powdery mildews.

## Conclusions

In conclusion, this work describes the successful transformation of *P. flocculosa* through a CRISPR-Cas9 approach to better understand its elusive mode of action as a BCA. This led to the confirmation of a functional role of the effector Pf2826 in the biocontrol activity of the BCA, thus expanding the realm of modes of action of these organisms.

## Methods

### Strains and growth conditions

Cultures were maintained and sporidia produced according to Santhanam et al. (16). Briefly, freeze-dried samples of *Pseudozyma flocculosa* stock cultures were transferred onto petri dishes containing YMPDA and allowed to grow for 5 days at 28 °C. Sporidium production was done by transferring 15 agar blocks from actively growing colonies into YMPD broth. Cultures were incubated at 28 °C on a rotary shaker at 150 rpm for 3 days.

### sgRNA synthesis and RNP complex formation

For targeting *Pf2826* gene, crRNA (173) was designed using the E-CRISP online tool (http://www.e-crisp.org/E-CRISP/) (Additional file 1: Fig. S1A). Corresponding DNAs were synthesized as single-stranded oligos (Life Technologies, CA) and were used as template for the sgRNA synthesis. EnGen sgRNA synthesis kit (NEB #E3322) from NEB (MA) was used to synthesize sgRNA, according to the manufacturer’s instructions. Resulting sgRNAs were purified and concentrated using RNA clean and concentrator-25 kit (#R1017) from Zymo Research (CA). Cas9 was purchased as purified protein from GenScript (#Z03386) and complexed with freshly prepared sgRNA following the manufacturer’s instructions. The resulting ribonucleoprotein (RNP) complex was used for in vitro cleavage assay and for the PEG-mediated transformation to target *pf2826* gene.

### In vitro digestion assay

For the in vitro cleavage assay, *Pf2826* gene was amplified from wild type *P. flocculosa* genomic DNA using Q5 polymerase (NEB # M0491S) with primers P1/P2 (Additional file 5: Table S3). PCR products were purified using QIAquick PCR purification kit (Qiagen, NL). Cleavage assay was performed by mixing 2 µl of Cas9 protein (300 ng/µl), 2 µl of 10 × Cas9 reaction buffer, 100 ng of freshly prepared sgRNA, and 200 ng of purified *Pf2826* PCR product, and the final volume was adjusted to 20 µl with sterilized Milli-Q water. The reaction mix was then incubated at 37 °C for 2 h in a water bath. Cleavage efficiency was analyzed on 1% agarose gel (Additional file 1: Fig. S1B).

### Protoplast preparation and PEG-mediated transformation

Protoplasts were generated according to Cheng et al. [27] and Santhanam et al. [17]. To generate Cas9-based knockout, 200 µl of protoplasts, 5 µg of the plasmid pRB-Hyg, 50 µl of RNP complexes, and 50 µl of PEG solution (PEG 4000 60% w/v, Tris–HCl 50 mM, CaCl_2_ 50 mM) were added sequentially in a 50-ml Falcon tube and incubated at room temperature for 15 min. Thereafter, 800 µl of PEG 4000 solution was added to the wall of the tube, mixed gently, and incubated at room temperature for 20 min. For protoplast recovery, 5 ml of YMPD supplemented with 0.8 M sucrose was added to the protoplasts, and the solution was incubated at room temperature with gentle shaking for 3 h. After recovery, 45 ml of lukewarm (42 °C) regeneration medium (YMPDA with 0.8 M sucrose) containing 50 µg/ml of Hygromycin B (Invitrogen, CA) was added to the protoplasts and the final mixture was immediately plated. For effector localization experiments, 200 µl of protoplasts, 5 µg of the plasmid pRB-2826:mCherry, and 50 µl of PEG solution were mixed in a 50-ml Falcon tube and incubated at room temperature for 15 min. The remaining transformation steps were as mentioned above.

### High resolution melting curve analysis

Primers (HRM-2826-F/R) were designed to bind flanking regions of sgRNA binding site. The HRM curve analysis was performed on a magnetic induction cycler (MIC) qPCR instrument (Bio Molecular Systems, Australia) using Precision Melt Supermix (BioRad, #1,725,112, USA) in a 20-µL reaction with 1 × Precision Melt Supermix, each primer at concentration of 300 nM and 5 ng of genomic DNA. At least two technical replicates were performed for each sample. The qPCR amplification was performed with the following program: initial denaturation of 3 min at 95 °C, followed by 40 cycles of 95 °C for 10 s, 61 °C for 30 s, with fluorescence reading at the end of each extension step. The qPCR was followed by a melting program where the amplicon was heated 65 to 98 °C by ramping up the temperature at 0. 2 °C/s with a continuous signal acquisition. The HRM curve data (Additional file 1: Fig. S2) were analyzed using the in-built HRM analysis software (Bio Molecular Systems, Australia). To confirm mutants identified by HRM, *Pf2826* gene was amplified with Q5 High-Fidelity DNA Polymerase (NEB MA, USA) using primers Pf2826-F and Pf2826-R, resulting amplicons were purified and sequenced.

### Tripartite assays

Wild type, *Pf2826*^*AX*^ and* Pf2826*^*59X*^ mutant strains were grown in baffled flasks containing 100 ml of YMPD broth at 28 °C on a rotary shaker set at 150 rpm for 3 days. *Blumeria graminis* f. sp. *hordei* (Bgh) infected barley leaves were sprayed with either water or with a spore suspension (2 × 10^6^ spores/ml) of *P. flocculosa* wild type or *Pf2826*^*AX*^ or *Pf2826*^*59X*^ until runoff. The inoculated plants were left to evaporate at room temperature for 20 min and covered with transparent plastic bag to maintain a high humidity rate (> 90%). Plant tissues were examined at 24 and 36 hpi by stereomicroscopy and sampled for scanning electron microscopy (SEM). The biocontrol activity of wild type, *Pf2826*^*AX*^ and* Pf2826*^*59X*^, was rated on a scale of 0–5) as described previously [17] where 0 = no collapse of conidial chains; 1 = 1 to 20%; 2 = 21 to 40%; 3 = 41 to 60%; 4 = 61 to 80%; and 5 = 81 to 100%. The statistical significance was evaluated by Student’s *t* test. For each treatment, a minimum of 5 leaves were rated at three different sites and the experiment was repeated three times.

### Scanning electron microscopy

For scanning electron microscopy (SEM), leaf segments were fixed by immersing them in 100% MeOH for 10 min and further dehydrated 2 × 30 min in 100% EtOH. Subsequently, the critical drying point was carried out on samples previously mounted on aluminum stubs and finally, gold coating (20 nm) was done prior to SEM observations. Samples were observed on an SEM Inspect^tm^ F50 from FEI microscope (Hillsboro, OR).

### Plasmid construction and localization of Pf2826

*Pseudozyma flocculosa* elongation factor eEF3 promoter was used for the constitutive expression of Pf2826 tagged with mCherry. The localization cassette containing eEF3 promoter driving the expression of Pf02826 tagged with mCherry at C-terminal, followed by *GPD* terminator was synthesized (GenScript, USA) and cloned into the plasmid pRB-Hyg at PacI site using HiFi-Neb assembly Mix (NEB MA, USA) following the manufacturer’s instructions to generate pRB-2826:mCherry. Localization cassette was transformed in the protoplast of wild type *P. flocculosa* using PEG-mediated transformation method. Transformants exhibiting bright red color were selected for the tripartite assay on barley infected with powdery mildew. Samples were collected 24 hpi and thin sections were cut and collected on glass slides. Tissues were observed under Leica SP8 confocal microscope to localize the Pf2826 effector.

### Cloning, expression, and purification of effector Pf2826

A double 6 × His tag at N-terminal followed by the coding sequence of Pf2826 or Pf2382 without signal peptide was synthesized and cloned in the pET-21b expression vector (Genscript, USA). The vector was transformed into *E. coli* strain BL21-DE3. Selected colonies were grown overnight in 5 mL LB medium supplemented with 100 μg/mL Ampicillin. One milliliter of overnight cultures was inoculated to 100 mL of LB medium supplemented with antibiotics, and cells were grown to the density of 0.5–0.6 (OD600) and induced with 1 mM of IPTG and further grown at 37 °C with 180 rpm for 2 h. Cultures were pelleted by centrifugation at 3000* g*, flash frozen in liquid nitrogen, and stored at − 20 °C. Recombinant proteins containing His-tag were purified using Ni–NTA Agarose purification system (Thermo Fisher # R90115) following the manufacturer’s protocol (https://tools.thermofisher.com/content/sfs/manuals/ninta_system_man.pdf). For hybrid protocol, bacterial cell lysate was prepared under denaturing conditions and washed and eluted using native buffers to refolding the proteins. Bacterial pellets were resuspended in guanidinium lysis buffer, and the suspension was gently rotated for 10 min at room temperature. Cell lysate was sonicated the on ice with a 5-s pulse for three times. The lysate was centrifuged at 3000* g* for 15 min to separate the fractions and 5 μL of each fraction was used for SDS-PAGE analysis. Pf2826 and Pf2382 were present in the insoluble fraction. Eight milliliters of lysate was added to the purification column and allowed to bind for 30 min at room temperature on a rotating wheel. Resin was allowed to settle by gravity, and supernatant was removed by pipetting. Column was washed with denaturing binding buffer by resuspending the resin. After 2 min on the rotating wheel, the resin was allowed to settle by gravity and the supernatant was pipetted out. The column was washed one more time with denaturing binding buffer. The column was washed with 8 mL of native wash buffer as described above for three times. Pf2826 and Pf2382 were eluted with 12 mL of native elution buffer and concentrated using Amicon® Ultra-15 centrifugal filter units (Millipore #UFC901008).

### Preparation of protein extract from tripartite assay for pull-down

*Pseudozyma flocculosa* wild type strain was grown in baffled flask containing 100 ml of YMPD broth at 28 °C on a rotary shaker at 150 rpm for 3 days. Spore suspension (2 × 10^6^ spores/ml) was prepared and sprayed on *Blumeria graminis* f.sp. *hordei*-infected barley leaves until the solution runoff. The inoculated leaves were left to evaporate at room temperature for 20 min and placed in large petri dishes lined with sterile wet paper to maintain a high humidity. Leaf samples were collected 36 hpi. For total protein extraction, 2 g of leaves was ground in 5 mL of 50-mM Na-phosphate buffer (pH 8.0), 150 mM NaCl, 1 mM β-mercaptoethanol, 2 mM MgCl_2_, 5% glycerol, 1% PVPP (w/v) supplemented with 1/2 protease cocktail inhibitor EDTA free (Calbiochem), and 2 μL of benzonase nuclease (Sigma-Aldrich). The plant extract was incubated on ice for 15 min, centrifuged at 4000* g* for 5 min at 4 °C, followed by a second centrifugation at 12,000* g* for 15 min at 4 °C. The supernatant was filtered through a 0.45-μm syringe filter to remove insoluble particles.

### Pull-down assay and protein identification by mass spectrometry

All pull-down assays (summarized in Additional file 5: Table S4) were performed with two technical replicates. Interacting partners of the Pf2826 and Pf2382 were identified using Dynabeads™ His-Tag isolation and pulldown (Invitrogen # 10103D) following the manufacturer’s instructions. Finally, the samples were eluted with 100 μL of His-Elution Buffer. Eluted samples were run on 12% acrylamide/bis-acrylamide (37.5:1) tris-tricine SDS-PAGE mini gels (Mini-Protean III system, BioRad) and stained with colloidal Coomassie blue. Each lane was cut into pieces, transferred to 1.5-mL tubes and stored at 4 °C. In-gel digestion followed by LC–MS/MS were performed at Plateforme protéomique, CHU de Québec-Université Laval, CHUL, Quebec City, Canada.

### Selection, cloning, and Y2H validation

A list of proteins identified in the Pf2826 interactome and absent in the negative controls (no bait and His-tagged Pf2382) were used for further protein–protein interaction investigation. The results from the localization assays were used to guide the selection of the effector’s putative interactors for validating their possible targets using the yeast-two-hybrid (Y2H) methodology. The eight selected candidates were A0A287N0S4 (germin); A0A287RAV7 (HvPR-1a); N1J4Z2 (Sgk2), A0A383V2V0 (Unch-Protein), N1J6Z3 (CSEP0313), A0A383UML4 (Unch-effector), N1J908 (Putati-effector), and F2DJR4 (Chitinase). The coding sequences of the selected targets were retrieved from the National Center for Biotechnology Information—NCBI (https://www.ncbi.nlm.nih.gov/). Primers were designed to clone the targets’ full-length CDS, excluding the signal peptide in the case of secreted proteins (Additional file 1: Table. S3). Total RNA was extracted from leaf samples from the tripartite assay 24 hpi with *P. flocculosa* spores’ suspension using the RNEasy Mini Purification Kit (QIAGEN) and including DNAse treatment as per the manufacturer’s instructions. cDNA was synthesized using the High-Capacity cDNA Reverse Transcription Kit (Applied Biosystems) and used as template for the PCR amplifications. The genes from barley and Bgh were cloned into the vectors pGADT7 (AD-vector) and pGBKT7 (BD-vector) using restriction enzymes (Additional file 5: Table S3). Confirmation of cloning was performed by sequencing. For the Y2H, the Yeastmaker™ Yeast Transformation System 2 (Clontech) was used following the manufacturer’s instructions. The Gold Yeast Two-Hybrid strain was made competent and transformed with the plasmids containing the Pf2826 effector and the preys in different orientations. Bait vector pGBKT7-Pf2826 with Gal4 DNA-binding domain, together with the prey candidate interacting proteins with Gal4 activation domain or other way orientation, was co-transformed into the Y2Hgold strain (Clontech, USA) according to the Clontech Yeast Transformation protocol (Clontech, USA). The transformants were grown on non-selective media (DDO: SD/-Trp/-Leu) and selective media (QDO: SD/-Trp/-Leu/-His/-Ade) at 30 °C for 3–5 days. For each yeast transformation, three independent transformed colonies were used for interaction validation.

### Generation of *U. maydis* strain expressing Pf2826

To express the *P. flocculosa* effector Pf2826 in *U. maydis* strain FB1, we followed the cloning and transformation procedures outlined in Teichmann et al. [28]. Briefly, the strong constitutive Potef promoter was used to express *Pf2826* and a carboxin resistance (ip) cassette as a selectable marker in *U. maydis*. Coding sequence of *Pf2826* was amplified and introduced into p123 under the control of the strong Potef promoter using NEB Hi-Fi assembly following the manufacturer’s instructions (NEB, USA). Prior to integrative transformation into the ip locus of *U. maydis* strain FB1, plasmids were linearized with SspI. For selection of transformants, PDA plates containing 2 µg/ml carboxin were used. Mutants were confirmed by PCR amplification and validated by sequencing. Assessment of the biocontrol activity of *U. maydis* wild-type strain FB1 and two independent FB1 transformants overexpressing *Pf2826* strains was performed exactly like *P. flocculosa* tripartite assay.

## Supplementary Information

**Additional file 1: Fig. S1.** *In vitro* digestion of *Pf2826* from *Pseudozyma* *flocculosa* using the ribonucleoprotein complex. **A.** Diagram of crRNA-173 binding site in the coding region of *Pf2826*. sign indicates the orientation of target strand. **B.** Agarose gels after electrophoresis show *in vitro* digestion at 37 °C for 2 hours of *Pf2826* amplicons with or without sgRNA complexed with Cas9 protein. **Fig. S2.** Detection of successful CRISPR-Cas9 transformants using HRM analysis. Region surrounding the sgRNAbinding site were amplified from Cas9 transformants and wild type *Pseudozyma* *flocculosa*. Amplicons were subjected to high resolution melting curve analysis and difference curves displaying the difference in fluorescence were generated using Mic-qPCR software. The experiment is based on two technical replicates and repeated twice with similar results. **Fig. S3.** Confirmation of gene editing event. Coding sequence of *Pf2826* was amplified from wild type *Pseudozyma* *flocculosa* and two positive transformants selected based on HRM analysis were sequenced. **A**. Pairwise chromatogram alignment of wild type, *Pf2826*^*AX*^ and *Pf2826*^*59X*^ strains showing a single base deletion upstream to the PAM sequence in the mutant *Pf2826*^*AX*^. In the mutant *Pf2826*^*59X*^, 59 bases were deleted around the sgRNA binding site. **B**. Pairwise protein sequence alignment of Pf2826 in wild type and mutant strains. Frame shift mutation caused by the single base deletion and 59 bases deletion has resulted in premature termination of translation. **Fig. S4.** Functional analysis of 79 candidate proteins interacting with Pf2826 from *Pseudozyma* *flocculosa*. Gene Ontology term annotation analysis of 79 potential interactors of Pf2826 was performed using Blast2GO. The proteins were annotated for molecular functions, cellular components, and biological process. The distributions are shown in percentage.

**Additional file 2.**

**Additional file 3.**

**Additional file 4: Tables S1.** Potential interactors that are unique only to the Pf2826 were identified after removing the proteins present in negative controls.

**Additional file 5: Table S2.** List of proteins selected from pull-down assay for validation using Y2H assay. **Table S3.** List of primers used in this study. **Table S4.** List of baits and control used in the pull-down experiments.

**Additional file 6.** Biocontrol assessment data for wild type *P. flocculosa* and Cas9 transformants inoculated on powdery mildew-infected barley (associated to Fig. 2B).

**Additional file 7.** Biocontrol assessment data for wild type *U. maydis *and FB1 transformants expressing *Pf2826* inoculated on powdery mildew-infected barley (associated to Fig. 5B).

## Data Availability

All data generated or analyzed during this study are included in this published article and its supplementary and additional information files.

## Abbreviations

BCA: Biocontrol agent
Bgh: *Blumeria graminis* f. sp. *hordei*
Co-Ip: Co-immunoprecipitation
CRISPR: Clustered regularly interspaced short palindromic repeats
crRNA: Crispr RNA
CSEP: Candidate secreted effector proteins
eEF3: Translation elongation factor 3
KO: Knock-out
OE: Overexpression
PR-proteins: Pathogenesis related proteins
RNP: Ribonucleoprotein
SEM: Scanning electron microscopy
Y2H: Yeast two-hybrid

## References

[1] Hückelhoven R, Panstruga R (2011). Cell biology of the plant-powdery mildew interaction. Curr Opin Plant Biol.

[2] O’Connell RJ, Panstruga R (2006). Tête à tête inside a plant cell: establishing compatibility between plants and biotrophic fungi and oomycetes. New Phytol.

[3] Pedersen C, van Themaat EVL, McGuffin LJ, Abbott JC, Burgis TA, Barton G (2012). Structure and evolution of barley powdery mildew effector candidates. BMC Genomics.

[4] Lu X, Kracher B, Saur IML, Bauer S, Ellwood SR, Wise R (2016). Allelic barley MLA immune receptors recognize sequence-unrelated avirulence effectors of the powdery mildew pathogen. Proc Natl Acad Sci U S A.

[5] Pennington HG, Gheorghe DM, Damerum A, Pliego C, Spanu PD, Cramer R (2016). Interactions between the powdery mildew effector BEC1054 and barley proteins identify candidate host targets. J Proteome Res.

[6] Mukhtar MS, Carvunis A, Dreze M, Epple P, Steinbrenner J, Moore J (2011). Plant immune system network. Science (80-).

[7] Weßling R, Epple P, Altmann S, He Y, Yang L, Henz SR (2014). Convergent targeting of a common host protein-network by pathogen effectors from three kingdoms of life. Cell Host Microbe.

[8] Ceulemans E, Ibrahim HMM, De Coninck B, Goossens A (2021). Pathogen effectors: exploiting the promiscuity of plant signaling hubs. Trends Plant Sci.

[9] Mukhtar MS, Carvunis AR, Dreze M, Epple P, Steinbrenner J, Moore J (2011). Independently evolved virulence effectors converge onto hubs in a plant immune system network. Science (80-).

[10] Bélanger RR, Labbé C, Lefebvre F, Teichmann B (2012). Mode of action of biocontrol agents: All that glitters is not gold. Can J Plant Pathol.

[11] Cheng Y, McNally DJ, Labbé C, Voyer N, Belzile F, Bélanger RR (2003). Insertional mutagenesis of a fungal biocontrol agent led to discovery of a rare cellobiose lipid with antifungal activity. Appl Environ Microbiol.

[12] Mimee B, Labbé C, Pelletier R, Bélanger RR (2005). Antifungal activity of flocculosin, a novel glycolipid isolated from *Pseudozyma flocculosa*. Antimicrob Agents Chemother.

[13] Mimee B, Pelletier R, Bélanger RR (2009). In vitro antibacterial activity and antifungal mode of action of flocculosin, a membrane-active cellobiose lipid. J Appl Microbiol.

[14] Mimee B, Labbé C, Bélanger RR (2009). Catabolism of flocculosin, an antimicrobial metabolite produced by *Pseudozyma flocculosa*. Glycobiology.

[15] Clément-Mathieu G, Chain F, Marchand G, Bélanger RR (2008). Leaf and powdery mildew colonization by glycolipid-producing Pseudozyma species. Fungal Ecol.

[16] Hammami W, Castro CQ, Rémus-Borel W, Labbé C, Bélanger RR (2011). Ecological basis of the interaction between *Pseudozyma flocculosa* and powdery mildew fungi. Appl Environ Microbiol.

[17] Santhanam P, Labbé C, Fietto LG, Bélanger RR. A reassessment of flocculosin-mediated biocontrol activity of *Pseudozyma flocculosa* through CRISPR/Cas9 gene editing. Fungal Genet Biol. 2021;153:103573. 10.1016/j.fgb.2021.103573. ISSN 1087-1845. 10.1016/j.fgb.2021.103573 34029708

[18] Laur J, Ramakrishnan GB, Labbé C, Lefebvre F, Spanu PD, Bélanger RR (2018). Effectors involved in fungal–fungal interaction lead to a rare phenomenon of hyperbiotrophy in the tritrophic system biocontrol agent–powdery mildew–plant. New Phytol.

[19] Lefebvre F, Joly DL, Labbe C, Teichmann B, Linning R, Belzile F (2013). The transition from a phytopathogenic smut ancestor to an anamorphic biocontrol agent deciphered by comparative whole-genome analysis. Plant Cell.

[20] Kim S, Kim D, Cho SW, Kim J, Kim J-S (2014). Highly efficient RNA-guided genome editing in human cells via delivery of purified Cas9 ribonucleoproteins. Genome Res.

[21] Brückner A, Polge C, Lentze N, Auerbach D, Schlattner U (2009). Yeast two-hybrid, a powerful tool for systems biology. Int J Mol Sci.

[22] Van Loon LC, Rep M, Pieterse CMJ (2006). Significance of inducible defense-related proteins in infected plants. Annu Rev Phytopathol.

[23] Gjetting T, Hagedorn PH, Schweizer P, Thordal-Christensen H, Carver TLW, Lyngkjær MF (2007). Single-cell transcript profiling of barley attacked by the powdery mildew fungus. Mol Plant Microbe Interact.

[24] Aguilar GB, Pedersen C, Thordal-Christensen H (2016). Identification of eight effector candidate genes involved in early aggressiveness of the barley powdery mildew fungus. Plant Pathol.

[25] Haskins RH, Thorn JA (1951). Biochemistry of the ustilaginales: VII. Antibiotic activity of ustilagic acid. Can J Bot..

[26] Teichmann B, Lefebvre F, Labbé C, Bölker M, Linne U, Bélanger RR (2011). Beta hydroxylation of glycolipids from *Ustilago maydis* and *Pseudozyma flocculosa* by an NADPH-dependent β-hydroxylase. Appl Environ Microbiol.

[27] Cheng Y, Bélanger RR (2000). Protoplast preparation and regeneration from spores of the biocontrol fungus *Pseudozyma flocculosa*. FEMS Microbiol Lett.

[28] Teichmann B, Labbé C, Lefebvre F, Bölker M, Linne U, Bélanger RR (2011). Identification of a biosynthesis gene cluster for flocculosin a cellobiose lipid produced by the biocontrol agent *Pseudozyma flocculosa*. Mol Microbiol.

