# Supplementary material for: A unique effector secreted by Pseudozyma flocculosa mediates its biocontrol activity
Source: BMC Biol. 2023 May 24;21:118. doi: 10.1186/s12915-023-01624-z (PMC10210494; doi:10.1186/s12915-023-01624-z)

Additional File 1: Fig. S1

A

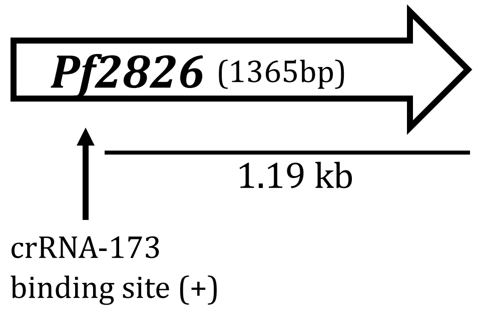

B

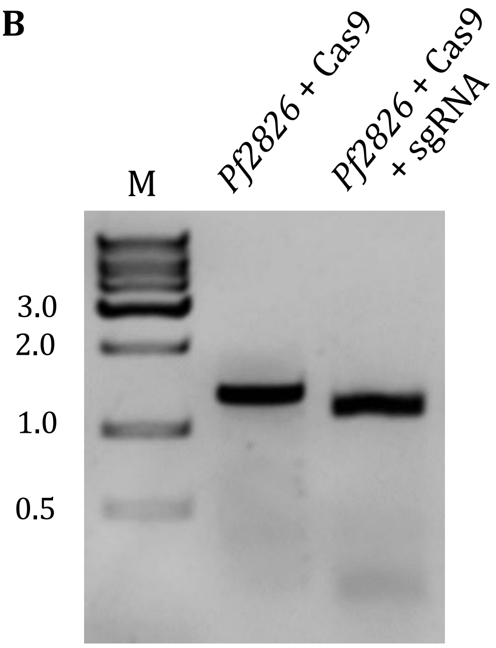

Additional file 1: Fig. S2

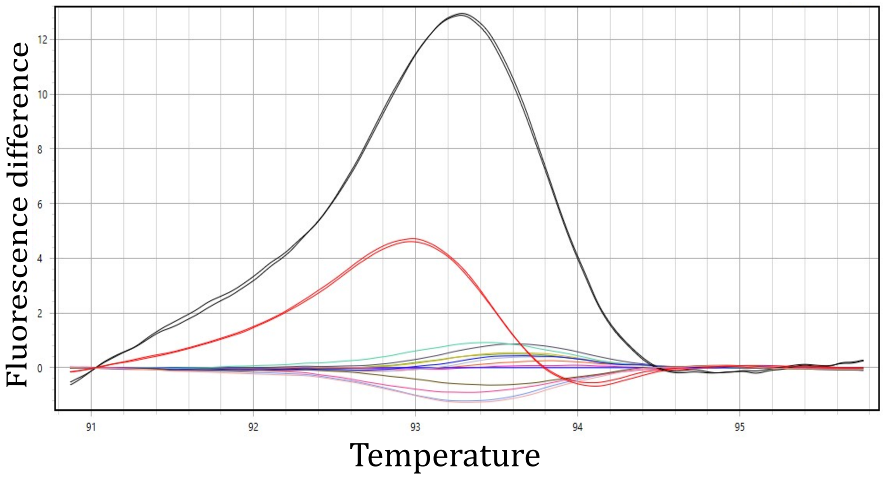

Additional file 1: Fig. S3

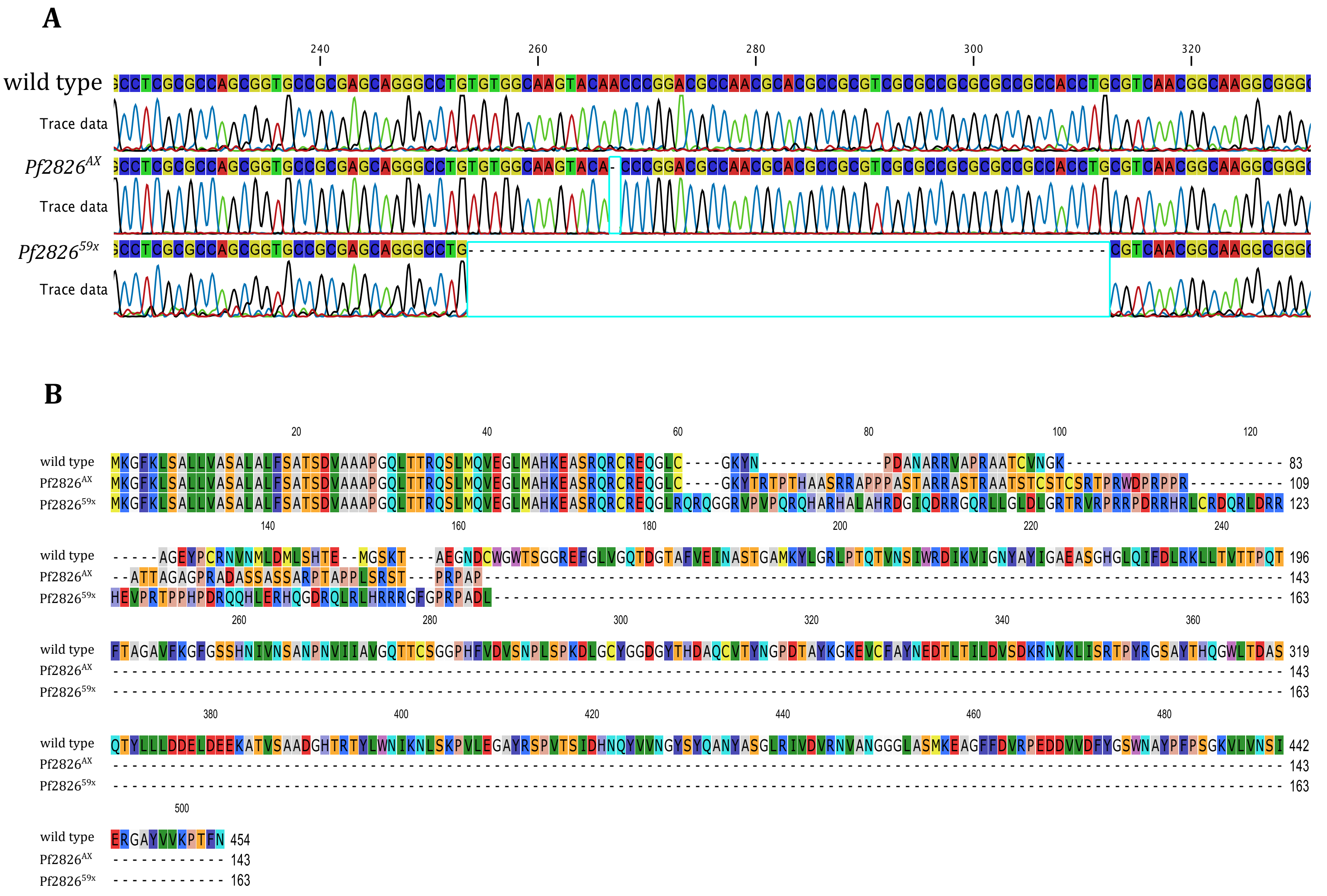

Additional file 1: Fig. S4

Molecular functions

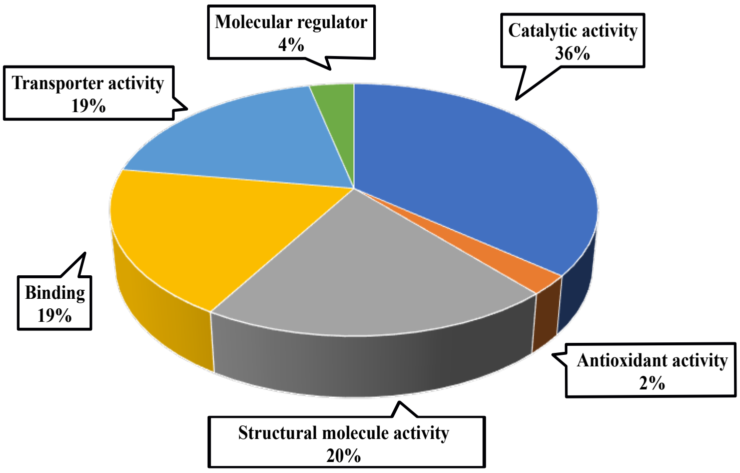

Cellular Components

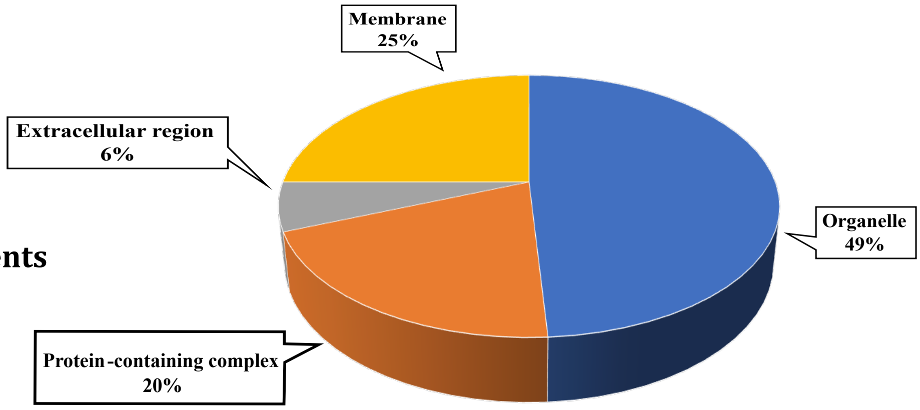

Biological process

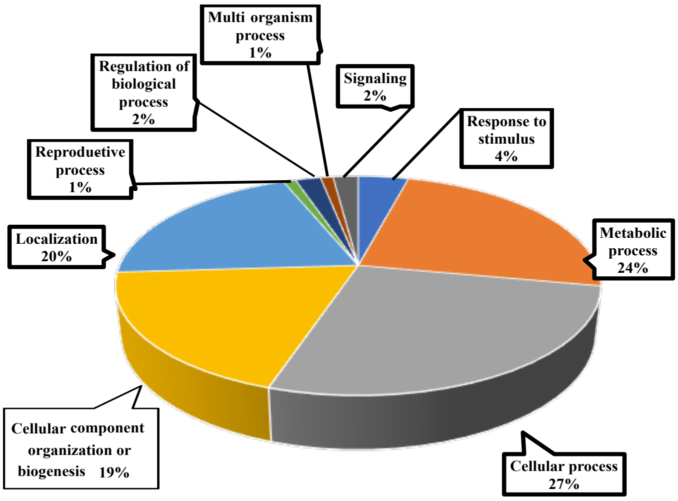

Supplement: Supplementary file 1 — Additional file 1: Fig. S1. In vitro digestion of Pf2826 from Pseudozyma flocculosa using the ribonucleoprotein complex. A. Diagram of crRNA-173 binding site in the coding region of Pf2826. sign indicates the orientation of target strand. B. Agarose gels after electrophoresis show in vitro digestion at 37 °C for 2 hours of Pf2826 amplicons with or without sgRNA complexed with Cas9 protein. Fig. S2. Detection of successful CRISPR-Cas9 transformants using HRM analysis. Region surrounding the sgRNAbinding site were amplified from Cas9 transformants and wild type Pseudozyma flocculosa. Amplicons were subjected to high resolution melting curve analysis and difference curves displaying the difference in fluorescence were generated using Mic-qPCR software. The experiment is based on two technical replicates and repeated twice with similar results. Fig. S3. Confirmation of gene editing event. Coding sequence of Pf2826 was amplified from wild type Pseudozyma flocculosa and two positive transformants selected based on HRM analysis were sequenced. A. Pairwise chromatogram alignment of wild type, Pf2826AX and Pf282659X strains showing a single base deletion upstream to the PAM sequence in the mutant Pf2826AX. In the mutant Pf282659X, 59 bases were deleted around the sgRNA binding site. B. Pairwise protein sequence alignment of Pf2826 in wild type and mutant strains. Frame shift mutation caused by the single base deletion and 59 bases deletion has resulted in premature termination of translation. Fig. S4. Functional analysis of 79 candidate proteins interacting with Pf2826 from Pseudozyma flocculosa. Gene Ontology term annotation analysis of 79 potential interactors of Pf2826 was performed using Blast2GO. The proteins were annotated for molecular functions, cellular components, and biological process. The distributions are shown in percentage. [file 12915_2023_1624_MOESM1_ESM.pdf]
