# Supplementary material for: A unique effector secreted by Pseudozyma flocculosa mediates its biocontrol activity
Source: BMC Biol. 2023 May 24;21:118. doi: 10.1186/s12915-023-01624-z (PMC10210494; doi:10.1186/s12915-023-01624-z)
Supplement: Supplementary file 5 — Additional file 5: Table S2. List of proteins selected from pull-down assay for validation using Y2H assay. Table S3. List of primers used in this study. Table S4. List of baits and control used in the pull-down experiments. [file 12915_2023_1624_MOESM5_ESM.pdf]

Additional file 5: Table S2. List of potential interacting proteins selected from pull-down assay for validation using Y2H assay. *Hordeum vulgare* subsp. *vulgare* (*Hvv*); *Blumeria graminis f. sp. hordei* (*Bgh*).

Uniport id	Protein names	Organism	Length aa	PCR	Y2H analysis
A0A287N0S4	Germin-like	*Hvv*	183	yes	yes
A0A287RAV7	HvPR-1a	*Hvv*	170	yes	yes
F2DJR4	Chitinase	*Hvv*	256	yes	yes
N1J4Z2	Sgk2 (Serine/threonine-protein kinase)	*Bgh*	234	yes	yes
A0A383UML4	Uncharacterized protein	*Bgh*	171	yes	yes
N1J6Z3	CSEP0313 (putative effector)	*Bgh*	384	yes	yes
A0A383V2V0	Uncharacterized protein	*Bgh*	511	yes	yes
N1J908	Putative candidate secreted effector	*Bgh*	134	yes	yes

Additional file 5: Table S3. List of primers used in this study.

Uniprot id	Primer id	Primer sequence	Size (bp)	Description
N/A	HRM-2826-F	GAGCCTGATGCAGGTCGAA	120	HRM analysis
	HRM-2826-R	CCTTGCCGTTGACGCAGGTG		
N/A	Pf2826-F	ATGAAGGGATTCAAGCTCAGC	1365	Sequencing
	Pf2826-R	CTAGTTGAAGGTCGGCTTGAC		
A0A287N0S4	Germin_NdeI_F	CACTG**CATATG**ACCGACCCTGACCCTCTAC	606	Cloning in pGADT7 and pGBKT7
	Germin_XmaI_R	CACTG**CCCGGG**TTAAGACCCACCGGCGAAC		
A0A287RAV7	HvPr1a_NdeI_F	ATCTG**CATATG**CAAAACTCGCCTCAGGACTAC	423	Cloning in pGADT7 and pGBKT7
	HvPr1a _XmaI_R	ATCTG**CCCGGG**TTAGTATGGTTTCTGTCCAACAACA		
N1J4Z2	Sgk2_NdeI_F	ATCTG**CATATG**ATGTCTGTTTCTCCAGAGG	705	Cloning in pGADT7 and pGBKT7
	Sgk2_XmaI_R	ATCTG**CCCGGG**TTATCCTAAAGTTTTTTCTTCATCT		
A0A383V2V0	Unch_protein_BamHI_F	ATCTG**GGATCC**AAATGTTGGACAGCCATCAAT	1536	Cloning in pGADT7
	Unch_protein_XhoI_R	ATCTG**CTCGAG**TTAATAAACTCCCATAATATCCTCA		
	Unch_protein_NcoI_F	ATCTG**CCATGG**AAATGTTGGACAGCCATCAAT		Cloning in pGBKT7
	Unch_protein_BamHI_R	CCTCC**GGATCC**TTAATAAACTCCCATAATATCCTCA		
N1J6Z3	CSEP0313_NdeI_F	ATCTG**CATATG**GAGGATTCCGTGTCAACAAGC	1098	Cloning in pGADT7 and pGBKT7
	CSEP0313_XmaI_R	ATCTG**CCCGGG**TCAGGTGATGTCGTTTACTCGA		
A0A383UML4	Unch_effector_BamHI_F	ATCTG**GGATCC**AAATGTACTGTGTGCCGCCCTCATC	462	Cloning in pGADT7
	Unch_effector_XhoI_R	ATCTG**CTCGAG**TTAAAAAATACGGATGGGAGTTTT		
	Unch_effector _NcoI_F	ATCTG**CCATGG**AAATGTACTGTGTGCCGCCCTCATC		Cloning in pGBKT7
	Unch_effector _BamHI_R	ATCTG**GGATCC**TTAAAAAATACGGATGGGAGTTTT		
N1J908	Putati_effector_NdeI_F	ATCTG**CATATG**GTGTCAGTTCCGAATAGTGGA	342	Cloning in pGADT7 and pGBKT7
	Putati_effector_XmaI_R	ATCTG**CCCGGG**TTAATTGAGTGAGCAGGCTCTGA		
F2DJR4	Chitinase_NdeI_F	GAGAGA**CATATG**ATGCAATCGGTGTACGCGAGCATG	768	Cloning in pGADT7 and pGBKT7
	Chitinase_BamHI_R	GAGAGA**GGATCCC**TAGCTAGCGAAGTTTCGCTG		

Additional file 5: Table S4. List of baits and control used in the pull-down experiments. Experiment was performed with two technical replicates.

Pull down-bait	Purified His-tagged Pf2826
Negative control-1	No bait
Negative control-2	Purified His-tagged Pf2328
